# Early marriage and women’s empowerment: the case of child-brides in Amhara National Regional State, Ethiopia

**DOI:** 10.1186/s12914-020-00249-5

**Published:** 2020-12-14

**Authors:** Mikyas Abera, Ansha Nega, Yifokire Tefera, Abebaw Addis Gelagay

**Affiliations:** 1 Department of Sociology, University of Gondar, Gondar, Ethiopia; 2 School of Public Health, Addis Ababa University, Addis Ababa, Ethiopia; 3 Institute of Public Health, University of Gondar, Gondar, Ethiopia

**Keywords:** Early marriage, Child-bride, Household, Decision-making, Systems theory

## Abstract

**Background:**

Women, especially those who marry as children, experience various forms and degrees of exclusion and discrimination. Early marriage is a harmful traditional practice that continues to affect millions around the world. Though it has declined over the years, it is still pervasive in developing countries. In Ethiopia, Amhara National Regional State (or alternatively Amhara region) hosts the largest share of child-brides in the country. This study aimed at assessing the effects of early marriage on its survivors’ life conditions – specifically, empowerment and household decision-making – in western Amhara.

**Methods:**

This study employed community-based cross-sectional study design. It adopted mixed method approach – survey, in-depth interview and focus group discussion (FGD) – to collect, analyse and interpret data on early marriage and its effects on household decision-making processes. The survey covered 1278 randomly selected respondents, and 14FGDs and 6 in-depth interviews were conducted. Statistical procedures – frequency distribution, Chi-square, logistic regression – were used to test, compare and establish associations between survey results on women empowerment for two groups of married women based on age at first marriage i.e., below 18 and at/after 18. Narratives and analytical descriptions were integrated to substantiate and/or explain observed quantitative results, or generate contextual themes.

**Results:**

This study reported that women married at/after 18 were more involved in household decision-making processes than child-brides. Child-brides were more likely to experience various forms of spousal abuse and violence in married life. The study results illustrated how individual-level changes, mainly driven by age at first marriage, interplay with structural factors to define the changing status and roles of married women in the household and community.

**Conclusion:**

Age at first marriage significantly affected empowerment at household level, and women benefited significantly from delaying marriage. Increase in age did not automatically and unilaterally empowered women in marriage, however, since age entails a cultural definition of one’s position in society and its institutions. We recommend further research to focus on the nexus between the household and the social-structural forms that manifest at individual and community levels, and draw insights to promote women’s wellbeing and emancipation.

**Supplementary Information:**

The online version contains supplementary material available at 10.1186/s12914-020-00249-5.

## Background

Early marriage is any marriage entered into before one reaches the legal age of 18 [1–3]. Though both boys and girls could marry early, the norm in many countries around the world is that more girls than boys marry young and someone older [3]. In Mauritania and Nigeria, for instance, “more than half of married girls aged 15-19 have husbands who are 10 or more years older than they are” [3].

Resilient and interlinked socioeconomic and normative factors (e.g. poverty, illiteracy, traditionalism, patriarchy, etc.) undermine women’s status, capabilities and choices, and ensure early marriage continues unabated in many developing countries [4]. As a harmful traditional practice, though it is more common in developing than developed countries, there are substantial variations between and within regions of the world and countries [3, 5–7]. For instance, half of the world’s child-brides live in South Asia; and, while early marriage is still most common in Sub-Saharan Africa, between them, these two regions host the 10 countries with the highest rates of early marriage [3, 5, 8].

By early 2000s, 59% of Ethiopian girls were marrying before 18 [9].^1^ As in the rest of Sub-Saharan Africa, early marriage in Ethiopia is gendered with only 9% of men aged 25–49 been married by 18 [10, 11]. Its effects are diverse and wide-ranging [3, 4]. In its onset, early marriage effectively ends childhood by limiting its victims’ opportunities for schooling, skills acquisition, personal development and even mobility. It also increases the risks of early onset of sex, adolescent pregnancy and childbearing, etc. [12, 13] whose negative outcomes are amplified by girls’ undeveloped physique and lack of or inadequate knowledge on healthy sexual and reproductive behaviours. Cumulatively, these effects of early marriage undermine girls’ and young women’s health, psychosocial wellbeing and overall quality of life [14–16].

Early marriage is not only a serious public health issue. It also exacerbates domestic violence [17] and undermines women’s status and decision-making powers [18, 19]. It increases women’s risk of intimate partner sexual violence, for it is built on spousal age gap, power imbalance, social isolation and lack of female autonomy. Globally, some 30% of girls (aged 15–19) experience violence by partners [20]. Bangladeshi women married during their adolescence, for instance, are subject to increased domestic violence and loss of autonomy, which, nonetheless, improved with their educational attainment [21]. Child-brides, specifically, are twice as likely as adult-brides to experience domestic violence [22]. This is partly because child-brides are more likely to be uneducated, poor and adherents to traditional gender norms [3, 23–26].

Child-brides are mostly isolated with restricted mobility and limited opportunities for independent living. Those who had been going to school would be coerced to discontinue when they marry, and those who have not been to school, the hope to do so dies on their wedding day. In Tach-Gaynt Woreda of Amhara region, for instance, 69% of young women marry early. Between 2009 and 2014, females represented 61% primary school dropouts in the Woreda; and, 34% of female school dropouts mentioned early marriage as the main reason. If child-brides want to start/continue schooling, a rare approval must come from husbands and/or families. In rural communities of Ethiopia, including Amhara region, the ‘good wife’ is primarily pictured in terms of what she accomplishes at home and for the husband, children and the elderly in the family and kinship.

Against the backdrop of mounting calls for legal and policy changes, Ethiopia introduced provisions [10] to redress gender inequalities and discrimination in its most recent Constitution (1995; Article 35:3) [27]; it has also revised its Family (2000) and Penal (2005) Codes to, among other things, raise the age of legal consent for women to 18 (from 15). Ethiopia’s latest Education and Training Policy [28] introduced provisions to reorient societal attitude towards and valuation of women in education, training and development. More profoundly, and partly due to international pressure, in 2013, Ethiopia spelled out its commitment to eradicate early marriage by 2025 in the *National Strategy and Action Plan on Harmful Traditional Practices against Women* [29]. These and other relevant documents informed governmental and nongovernmental interventions to remove barriers, including early marriage, to young women’s personal advancement and empowerment, and taking effect at individual, institutional, national and cultural levels.

Accordingly, age at first marriage has been increasing over the years in Ethiopia [9]; nonetheless, its reported scale and rate are suspect for two main reasons. First, the Ethiopian Demographic and Health Survey (EDHS) defines age at first marriage as the age at which partners begin living together under one roof [29], despite the fact that many early marriages in Ethiopia allow spouses to start living together only a few years later as in the cases of promissory or child marriages [4]. Second, systematic underreporting or omission is a high possibility, which would lower the magnitude of early marriage among girls than boys as the latter commonly delay marriage. Criminal prosecution under the Revised Family Code (Article 7) could also induce underreporting or deliberate omission of early marriages.

Though there needs to be caution in interpreting statistics on early marriage in Ethiopia, it has been amply documented that Ethiopian women’s low social status explains their limited rights and odds to assume duties, roles and authority on equal terms as their male counterparts [9, 30]. Early marriage, one manifestation of this violence, is intimately linked with gender, poverty and illiteracy in rural Ethiopia [30]. Rural women tend to marry younger than those in urban areas, while patriarchy and the feminization of poverty, illiteracy and low educational attainment play crucial role in perpetuating the imbalance [9, 30].

There are studies that document strong association between early marriage and poverty. UNICEF reports that one in three girls in low- to middle-income countries will marry before 18 [3, 31]. Nonetheless, though many see a strong link between poverty and early marriage, the correlation is never monotonic. Family riches are not guarantee to avoid early marriage. With growing population and land shortages, girls from better-off families who stand to inherit valuable resources have become easy targets for sustained solicitations by those who desire to ‘marry-into’ wealth. Conversely, poor families generally resort to early marriage as a strategy to reduce economic vulnerability. In both scenarios, however, early marriage is seen as a mechanism to strengthen ties between families, evade the risk of daughters engage in premarital sex (and lose their virginity and/or become pregnant) or pass the culturally defined ‘desirable age’ for marriage (and become unmarriable).

The sociocultural consequences of becoming pregnant outside wedlock are harsh as they go against deep-rooted cultural norms that tie girls’ chastity and sexual purity before marriage to their family honor as well as their marriageability. Most parents fear delaying marriage makes sexual encounters imminent – consented or otherwise – that disgraces the family and tarnishes girls’ reputation and, subsequently, marriage prospects.

Within Ethiopia, girls in some regional states are more likely to marry early; and, Amhara region has the highest prevalence of early marriage with 50% of girls marrying at 15, and 80% marrying at 18 [32, 33]. In 2014, 74% of women [20–24] in the region married before 18, significantly higher than the national average of 41% [2]. To put this in perspective, “a girl born in [Amhara region] is three times as likely as the girl born in Addis Ababa to marry early” [3].

Reports on improving inter-generational age at first marriage at national level puts the persistently high prevalence of early marriage in Amhara region in a curious light [34]. In the region, early marriage is deeply entrenched in religious and cultural norms where sex before marriage is a blow to a girl’s marriageability, for her worth lies in her sexual purity, her future role as a devout wife and mother, and her commitment to family honor [35]. Hence, despite proactive laws, institutional structures and project interventions, early marriage grew adept and continues to affect the lives of many under different guises.

Due to its myriad nature [36, 37], on the other hand, eradicating early marriage requires simultaneously addressing its various dimensions and promoting girls’ empowerment through education, institutional support structures and community development programs. Informed by a mixed-methods approach, thus, this study aimed at informing such types of interventions at national and regional levels by identifying its association with women’s empowerment at three Zones (North Gondar, South Gondar and West Gojjam) of Amhara region – the regional State with “one the world’s highest rates of child marriage” (and the highest in Ethiopia) where “most unions take place without girls consent” [38]. The effects of early marriage go beyond the child-brides and their children, for they severely undermine national and global progress on a variety of Sustainable Development Goals, i.e., Agenda-2030. In light of this, this interdisciplinary study, falls within the current research priority agenda of promoting evidence-based policymaking and interventions [39] to mitigate early marriage as a resilient sociocultural problem – both from a human rights standpoint and meeting the Sustainable Development Goals targets.

Theoretically, systems theory, with its roots in Ludwig von Bertalanffy’s general systems theory, informs this study [40–42]. General systems theory argues that all entities – physical, biological, chemical, social, etc. – are complex, structured and dynamic systems, and they constitute sub-systems or units that interact with one another as well as the external environment. His theory advanced remarkably over the years with applications in biology [43–45], economics [46–48], psychiatry [49, 50] and sociology [51–54], among others.

In the field of family studies, systems theory has been used to study family or marriage as an interactional system, whereby patterns in members’ behaviors reflect interdependencies and communications amongst each other and with their normative environment, primarily – rather than their idiosyncrasies. As such, it brings at least two advantages to the current study: firstly, it allows us to understand the norms that structure families, marital relations, individual choices and decisions; secondly, it helps us unravel the tensions between agency and structure i.e., how changes at individual, family and cultural levels feed on each other to make family or marriage a dynamic interactional system capable of recalibrating its functions, communications, etc. vis-à-vis subsystems other systems in its sociocultural milieu [55, 56].

Using systems theory, hence, this study explores the effects of early marriage on child-brides interactional outcomes of a series of factors, including individuals’ personal convictions, the function of marriage (for instance, marriage in traditional societies is primarily a cultural arrangement that decent groups use to cement desirable alliances), normative definitions of sex, sexuality, etc. In other words, this study will treat early marriage as part of a broader, normative system where decisions or actions cannot be random but aim to create, maintain or re-create a state of equilibrium. Consensus, conflict, abuse or violence in a family, as Stratus puts it, can viewed as, primarily, products of the system than individual pathology [55]. Factors that perpetuate any of these scenarios in a family are embedded within the very fabric of the culture and norm that structure the family institution and relations among members i.e., individuals cannot randomly opt out of the norms of the system patterns without suffering consequences for their indiscretions or violations.

## Methods

### Description of the study area

The Amhara region is one of the 10 regional states and 2 city administrations that make-up the Federal Democratic Republic of Ethiopia.^2^ The region has an estimated population of 21.13million, with 90.85% residing in rural areas. Agriculture is the mainstay of residents in rural areas, with tourism, services and commerce creating the majority of jobs for urbanites. In 2013, Net Enrolment Rate at primary level was 93%, with gender parity at 0.95 [57]. The national adult literacy rate was 41.5% in 2012 [58].

This study covered 7 administrative districts – five *Woredas* and two cities – located in three Zones – North Gondar,^3^ South Gondar, West Gojjam – of northwestern Amhara region i.e., Chilga (Code.01), Gondar Zuria (Code.02), Libo-Kemkim (Code.05), Derra (Code.06) and Yèlma-èna-Dénsa (Code.07) *Woredas*, and cities of Gondar (Code.03) and Bahir Dar (Code.04). These districts are of varying sizes and they are subdivided into *Kebeles* – smallest administrative unit in the Ethiopian federal structure. The fieldwork was conducted between January and April 2017.

### Study population

This study covered all women who had had their first marriage within 10-years prior to the fieldwork, irrespective of their current marital status, in western Amhara region. The 10-years timeline provided a reasonably representative group of married women who would furnish sufficient data to assess changes in the incidence, prevalence and multifaceted effects of early marriage on their life conditions.

### Study design

This study employed a mixed method approach involving quantitative and qualitative methods. A cross-sectional study design with descriptive and analytical components enabled a comparative assessment of the effects of early marriage on women’s empowerment in the domestic sphere. Theoretically, system theory informs the discussion, analysis and interpretation of data i.e., by taking into account both individual (e.g., age) and ecological (e.g., cultural value, public policy) factors as they interact and affect actors’ behaviors (in this case, interpersonal interactions and decision-making) at household level.

### Methods of the study

Survey, focus group discussions (FGDs) and in-depth interviews generated relevant data on married women. A representative sample of 1278 married women were surveyed to gathered data on the prevalence and outcomes of early marriage in western Amhara region. Qualitative methods – FGD and in-depth interview – were used to assess married women’s experiences, community perceptions and values on (early) marriage, appropriate age of marriage, and impact of early marriage and community change-actors, among others. Critical desk-review of relevant documents generated perspectives and insights to triangulate the results of primary data.

### Sample size

Survey sample size was calculated using a single population formula, by assuming the proportion of early marriage in Ethiopia among married women whose age less than 24-years at 41% [2], with 95% confidence level and 4% margin of error: **581**. But after considering design effect for two-stage cluster sampling (*2) and non-response rate (*10%), the final survey sample size was determined at **1278** (=581*2 + (581*.10)).

To collect qualitative data, 2 types of FGDs were conducted in each of the 7 districts with, on average, 8 discussants: FGD_1_, with child-brides – a mixed length of age at first marriage i.e., 1–5 years and 6–10 years, and their residential place i.e., rural or urban; and, FGD_2_, with representatives of community leaders, elders, law enforcement officers, parents, school directors and governmental and non-governmental organizations working on children and girls. In total, 14 FGDs were conducted.

### Sampling procedure

Probability and purposive sampling techniques were used, respectively, for survey, and FGD and in-depth interview. Firstly, 7 districts – 5 *Woredas* (Chilga, Gondar Zuria, Derra, Libo-Kemkim and Yèlma-èna-Dénsa) and 2 cities (Gondar and Bahir Dar) – of Amhara region were identified, for they host community intervention projects intended to curb early marriage. Secondly, 4 *Kebeles* from each district were selected and the sampling procedure accounted for differences among districts in their residential pattern (urban vs. rural) and availability of community intervention projects (beneficiaries vs. non-beneficiaries). Specifically, the sampling procedure followed a 3:1 urban: rural ratio for the two cities, and the reverse for the 5 *Woredas*. Finally, the 1278 survey sample was distributed to each *Kebele* based on its population size and the number of women in reproductive age (ages, 15-49). Using *Kebele* residents’ rosters as sampling frame, a random – and proportionate – sample of households were selected for the survey from each *Kebele*.

### Data collection tools and procedure

All data collection tools (enumerator-administered questionnaire, and FGD and in-depth interview guides) were initially designed in English. They were translated into *Amharic,* and then back to English – forward-and-backward translation – to ensure their validity and consistency. The questionnaire was pilot-tested at *Teda Kebele* of North Gondar Zone, a *Kebele* excluded from the survey, to check for its validity, reliability and consistency. The pilot improved the questionnaire’s completeness, appropriateness, conciseness and relevance as well as the feasibility of the fieldwork.

Twenty-eight females were employed as survey enumerators from World Vision–Ethiopia’s roster of data collectors that documents trained, experienced, locally-resourceful youth for possible recruitment as enumerators, interviewers, guides, etc. in research projects. These enumerators and local guides underwent 2-days intensive training on research methods, data collection tools, interviewing skills, etc. including running mock-interview sessions. After the training, they administrated survey questionnaires by travelling from household to household. They, before asking survey questions, were required to explain the objective of the study, requested for informed consent to participate in the study and checked respondents’ profile for eligibility i.e., women married within 10 years during the fieldwork.

Two types of FGDs, 14 in total, were conducted: FGD_1_ involved child-brides who were identified and invited by enumerators during the survey; and, discussants for FGD_2_ were identified based on their knowledge of the problem of early marriage in the study area and approached via administrative channels. Finally, 6 in-depth interviews were conducted with child-brides, chosen purposively as their experiences vividly illustrate the effects of early marriage on women’s empowerment.

After inquiring about preferences and confirming with participants, FGDs and in-depth interviews were conducted in facilities and spaces convenient to all such as offices of World Vision–Ethiopia, Gender and Legal Affairs, and Youth Centers. These facilities and spaces were assessed beforehand for their cleanliness, calm, safety and accessibility as well as falling outside non-participants’ earshot and possible intrusions. On average, FGDs and in-depth interviews took, respectively, 60 and 40 min to complete. Authors conducted FGDs and interviewed child-brides.

### Data management and analysis

For the survey, all filled and returned questionnaires were checked for completeness and consistency of responses. Once survey data collection was finalized, 3 experienced data encoders entered questionnaire data into Epi-Info and later transferred to SPSS [20] as data-sets for cleaning, organization and analysis. Descriptive and inferential statistics were employed to determine, among others: the prevalence of early marriage; the incidences and magnitude of bad outcomes of early marriage on women’s decision-making; and, community’s perception on early marriage and appropriate age of marriage. Binary logistic regression models were used determine the likely occurrence of different forms of disempowerment in two groups of women i.e., those married before 18 and those married at/after 18. A *p*-value of 0.05 was used as a cut-off point to determine statistical significance.

Regarding FGDs and in-depth interviews, all sessions, with the consent of participants, were digitally recorded. Audio-files were later transcribed, post-coded and categorized under core thematic areas. Thematic content analysis provided insights into the nature, community perception and drivers of early marriage and changes. Analytical descriptions and quotes from FGDs and in-depth interviews were used to triangulate, contextualize or explain survey results. Narrated texts, graphs and tables were used to present results according to the nature of the information derived.

In quoting directly from FGDs and in-depth interviews, codes were used to refer to the method, source and location (districts). Accordingly, FGD-R01, for instance, refers to an FGD conducted with representatives of relevant stakeholders (i.e., R) in Chilga *Woreda* of North Gondar Zone (i.e., 01). Similarly, Interview-S07 refers to an interview conducted with child-brides (i.e., S) in Yèlma-èna-Dénsa *Woreda* of West Gojjam Zone (i.e., 07).

### Ethical considerations

Data for this article are taken from a larger study the authors conducted on behalf of *E*_*4*_*Y Project*, a project run by World Vision-Ethiopia and cleared for appropriate ethical standards at national and regional levels. On behalf of the authors, World Vision–Ethiopia supplied official letters to the respective regional and district administration offices and provide support and facilitation as required.

During the fieldwork, study participants and/or parents/legal guardians (when participants were under the age of 18) were informed about the study objectives and the scope of their involvement beforehand. Verbal consent was obtained from participants or parents/guardians prior to commencing survey, interviews or FGDs. Privacy and confidentiality were granted and maintained during the survey, discussions or interviews. Confidentiality of digital recordings and transcribed data were strictly protected and this was explained to all participants. During FGDs and in-depth interviews, special attention was given to when asking sensitive questions based on local contexts. Participants’ concerns and questions were addressed before they provided individual, informed consents. There was no financial incentive offered to study participants. Nonetheless, participants who had to travel from distant *Kebeles* for study’s purpose were provided with transport allowance.

The preliminary findings of the study were presented and validated in a national validation workshop held at Bahir Dar city (Ethiopia) and in attendance were representatives of the community (including study participants) and relevant governmental and non-governmental organizations working on early marriage. Workshop participants reflected on the process and results of the study. The authors addressed the comments and questions raised during the workshop, and they revised the study report submitted to World Vision-Ethiopia.

## Results

The results and findings of the study are organized and presented in two sub-sections: (a) the prevalence of early marriage; and (b) early marriage and household decision-making in Amhara region. Let us start with the prevalence of early marriage and its variation among districts of the region.

### The prevalence of early marriage in Amhara region

**Fig. 1:**
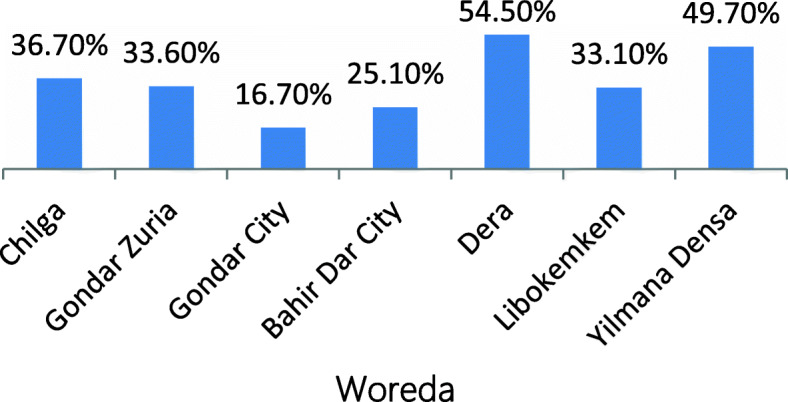
Prevalence of early marriage in Western Amhara, Ethiopia (Survey, 2017)

**Fig. 2:**
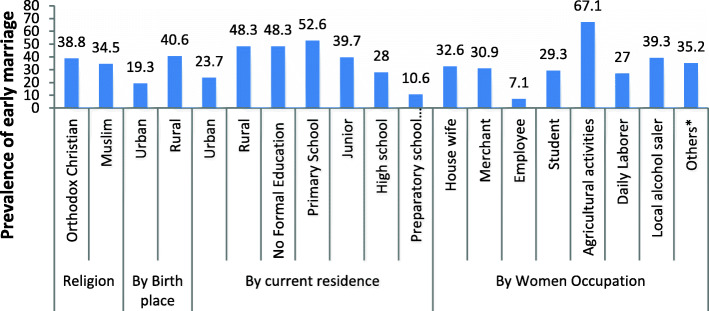
Prevalence and profile of early marriage in Amhara National Regional State, Ethiopia (Survey, 2017)

The survey covered 1278 married-women respondents, while 112 [6] participants took part in 14 FGDs (interviews). Of the 1278 respondents, 444 (34.7%) were married before the age of 18 Fig. 1. Nonetheless, as Fig. 2 reports, there was variation in the prevalence rate of early marriage among districts in the study area: Derra (54.5%) and Yèlma-èna-Dénsa (49.7%) *Woredas* registered the highest, and the cities of Gondar (16.7%) and Bahir Dar (25.1%) the lowest rates of early marriage. With the regional prevalence rate of 32%, the results indicated that urbanization is inversely related to the prevalence rate of early marriage.

Comparatively, early marriage was high among Orthodox Christians (38.8%) and rural residents (40.6%). Regarding schooling, the proportion of child-brides increased from ‘no formal schooling’ (48.3%) to ‘primary level’ (52.6%), before it declined at junior (39.7%) and senior (28%) high-school levels. These results underlined rural residents and primary grades as potent entry points for any effective intervention, for 53% of primary graders and 41% of rural residents ended up marrying before 18.

Respondents’ age at first marriage ranged from 5 to 35 (M = 18.75; SD = 3.44); and, the lowest ages to start living with spouses and make sexual debut among respondents were, respectively, 9 (M = 18.93; SD = 3.25) and 10 (M = 18.80; SD = 3.11).

Among respondents primarily engaged in farming, on the other hand, 67.1% experienced early marriage, which is not unexpected since the prevalence of early marriage is high in rural areas where agriculture is the main employer of labor. Similarly, 39.3% those who produce and sell local alcoholic beverages were married before 18 (Fig. 2). These and the results presented above indicate that early marriage has pertinent impacts on and associations with young women’s education, economic development and wellbeing.

### Early marriage and household decision-making in Amhara region

In this section, the effects of early marriage on young women’s empowerment at household decision-making processes are presented under five sections: early marriage, marital interactions and dysfunctions; early marriage and spouse abuse; early marriage and household management; early marriage, social interactions and procreation; and early marriage and healthcare.

#### Early marriage, marital relations and dysfunction

As Table 1 shows, respondents’ current living arrangement with first husband – which, though imperfectly, serves as a proxy to history of family dysfunction – significantly varies by their age at first marriage (χ^2^ = 34.296; α = .001). Family dysfunction refers to processes that undermine the intactness of the family institution and members’ ability to procreate, socialize children and support each other in life. These processes include, among others, conflict, abuse, role-strain, apathy, separation, divorce and desertion. In this study, when respondents did not share households with their first husbands at the time of the survey, it was taken to imply some form of family dysfunction i.e., conflict, abuse, separation, divorce, etc. Specifically, while 82.4% of the respondents married at/after 18 were living with their first husbands, only 68.2% of those married before 18 did. In other words, grim by-products of marriage such as separation, divorce, desertion (and possible remarriage) seems to be forced on women who had their first marriage before 18 – the legal age of consent under the Ethiopian Civil Code.

**Table 1:** Current living arrangement with first husband by age at first marriage

			Age at first marriage		Total
			< 18	≥18	
Currently living with first husband ****	No	N (%)	140 (32)	147 (18)	287 (23)
	Yes	N (%)	304 (68)	687 (82)	991 (77)
**Total**		**N (%)**	**444 (100)**	**834 (100)**	**1278 (100)**
Reason for not living with first husband*****	*Divorced & single*	N (%)	85 (19)	101 (12)	186 (15)
	*Divorced & remarried*	N (%)	42 (9)	21 (3)	63 (5)
	*Widowed*	N (%)	3 (1)	9 (1)	12 (1)
	*Inapplicable*	N (%)	314 (71)	703 (84)	1017 (80)
**Total**		**N (%)**	**444 (100)**	**834 (100)**	**1278 (100)**

Percent in cells is calculated *within* age at first marriage

****sig at α = .001 (Source: Survey, 2017)

To put it in context, a logit model predicts girls married before 18 are more than twice as likely (=*e*^0.137^) as women married at/after 18 not to be with their first husband (Logit: χ^2^ = 31.431; α = .001; Wald = 31.388; *ß* = .770; Constant = 772*)*. Significantly more respondents married before 18 also dissolved their first marriage and remarried (42 (9.5%)) than those married at/after 18 (21 (2.5%)). Specifically, girls married before 18 are twice as likely as women married at/after 18 to dissolve their first marriage, and either establish a new one or become widow or single (χ^2^ = 45.380; α = .001). For FGD participants at Libo Kemkim, these experiences tend to make the lives of child-brides grimmer:

Most of them [child-brides] would not have strong foundation to build their marriage on and end up being divorcees. After divorce, they migrate to urban areas and, due to lack of opportunities for education or employment, become street children or, worse, prostitutes. They are ghastly populating this cruel occupation. Many also migrate to Arab countries as divorce implies loss of livelihood [FGD_R06].

Mostly in rural communities of western Amhara region, underage girls enter into marriage without a personal, informed choice. For marriage generally is the result of the decision of parents and/or close kin, and it is culturally desirable for girls to marry men much older than themselves. But as they drop out of school and become child-mothers, several child-brides resented their husbands, parents and others who brokered and/or enabled the loss of their childhood:

It is a challenge to raise a child and taking care of household chores while still being a child! If I were to give birth now, I will be physically mature to take care of my duties effectively. I would have more time for myself too. I think marrying and giving birth as children have stunted our development … We do not lead a decent living and we do not cloth or clean up well. This is the result of our parents’ decision to marry us early …. [Moreover,] our children did not get the best we could have provided in care and protection. For lack of knowledge, we neglected them and this would not have happened if we married after we matured well enough. We do not clean them as required. Despite all this, we managed to see them grow. We do not want to see them grow repeating what we passed through, though. We want them to go to school, mature physically and mentally, enjoy life before they assume the responsibility of running a household the way we did/do [FGD_S05].

#### Early marriage and spouse abuse

Higher rates of first marriage dissolution, separation or desertion were not the only outcomes more likely associated with early marriage in the study population. Child-brides who remained married to their first husbands were highly vulnerable to spousal abuse and violence. Chi-square test of association (χ^2^ = 11.311; α = .01), for instance, found that child-brides were more likely to experience spousal verbal abuse (46.9%) than women married at/after 18 (36.9%). Specifically, women married at/after 18 are 33% (=*e*^0.119^) less likely to experience spousal verbal abuse than child-brides (Logit: χ^2^ = 11.247; α = .001; Wald = 11.261; *ß* = -.440; Constant = .797*)*. In a patriarchal society where both women and men accept some type of spousal abuse as a normality in marriage, the results show that delaying marriage until or past 18 was associated with small but statistically significant decline in spousal verbal abuse (Table 2).

**Table 2:** Dimensions of abuse by first husband by age at first marriage

			Age of first marriage		Total
			< 18	≥18	
Ever verbally abused by first husband***	No	N (%)	237 (53)	526 (63)	763 (60)
	Yes	N (%)	207 (47)	308 (37)	515 (40)
**Total**		**N (%)**	**444 (100)**	**834 (100)**	**1278 (100)**
Ever beaten by first husband***	No	N (%)	372 (84)	745 (89)	1117 (87)
	Yes	N (%)	72 (16)	89 (11)	161 (13)
**Total**		**N (%)**	**444 (100)**	**834 (100)**	**1278 (100)**
Ever forced for sex by first husband****	No	N (%)	319 (72)	716 (86)	1035 (81)
	Yes	N (%)	125 (28)	118 (14)	243 (19)
**Total**		**N (%)**	**444 (100)**	**834 (100)**	**1278 (100)**

Percent in cells is calculated *within* age at first marriage

****sig at α = .001; ***sig at α = .01 (Source: Survey, 2017)

Similarly, compared to those married at/after the legal age of 18, child-brides were also more likely to experience spousal beating (χ^2^ = 8.090; α = .01) and non-consensual sex or marital rape (χ^2^ = 36.903; α = .001) by their first husbands compared to woman married at/after 18. Specifically, women married at/after 18 were 38% (=*e*^0.171^) and 58% (=*e*^0.145^) less likely to experience spousal beating (Logit: χ^2^ = 7.845; α = .01; Wald = 7.986; *ß* = -.483; Constant = .694*)* spousal non-consensual sex (Logit: χ^2^ = 35.520; α = .001; Wald = 35.712; *ß* = -.866; Constant = .808*)*, respectively, as compared to child-brides.

#### Early marriage and household management

Child-brides were also more subservient/subordinate to their husbands in the administration of family possession and/or money (χ^2^ = 21.428; α = .001). While 45% of child-brides reported the main responsibility to administer family possessions and/or money rested in the husband, less than one-in-three women married at/after 18 reported similar scenarios. Furthermore, the percentage of respondents who share the responsibility of administering family resources with their husbands increased from 51.6 to 65% among those married before and at/after 18 respectively.

Similarly, child-brides’ decision-making roles in major family transactions and activities e.g., buy or sell land, livestock, groceries, children’s clothing, etc. were significantly lower than women married as adults (χ^2^ = 33.702; α = .001).

As a norm, Ethiopian women have the responsibility of taking care of family members including children, the elderly, etc. As Table 3 shows, decisions on how and when married women dispense with this role disproportionately involves husbands. Only 14 and 19% of respondents married before and at/after 18, respectively, were the main decision makers on buying groceries (χ^2^ = 14.608; α = .01); and, 2 and 3% of those married before and at/after 18, respectively, had made decisions on purchasing children’s clothing (χ^2^ = 10.799; α = .02). On a related note, collaborative decision-making on both issues *and* respondent age-categories improved at the expense of husbands’ share. Nonetheless, married women had better decision-making powers in purchasing groceries (17%) than children’s clothing (3%).

**Table 3:** Decision-making and administration of household affairs by age at first marriage

			Age of first marriage		Total
			< 18	≥18	
Administrator: Family possessions****	I, Myself	N (%)	14 (3)	27 (3)	41 (3)
	Husband	N (%)	201 (45)	269 (32)	470 (37)
	Together	N (%)	229 (52)	538 (65)	767 (60)
**Total**		**N (%)**	**444 (100)**	**834 (100)**	**1278 (100)**
Decision-maker: Major family matters****	I, Myself	N (%)	9 (2)	24 (3)	33 (3)
	Husband	N (%)	103 (23)	107 (13)	210 (16)
	Together	N (%)	256 (58)	601 (72)	857 (67)
	Inapplicable	N (%)	76 (17)	102 (12)	178 (14)
**Total**		**N (%)**	**444 (100)**	**834 (100)**	**1278 (100)**
Decision-maker: Buy groceries for family***	I, Myself	N (%)	61 (14)	161 (19)	222 (17)
	Husband	N (%)	59 (13)	77 (9)	136 (10)
	Together	N (%)	251 (56)	497 (60)	748 (58)
	Inapplicable	N (%)	73 (16)	99 (12)	172 (14)
**Total**		**N (%)**	**444 (100)**	**834 (100)**	**1278 (100)**
Decision Maker: Buy children’s clothing **	I, Myself	N (%)	7 (2)	27 (3)	34 (3)
	Husband	N (%)	58 (13)	75 (9)	133 (10)
	Together	N (%)	178 (40)	384 (46)	562 (44)
	Inapplicable	N (%)	201 (45)	348 (42)	549 (43)
**Total**		**N (%)**	**444 (100)**	**834 (100)**	**1278 (100)**

Percent in cells is calculated *within* age at first marriage

****sig at α = .001; ***sig at α = .01; **sig at α = .05 (Source: Survey, 2017)

#### Early marriage, social interactions and procreation

As Table 4 depicts, women married at/after 18 were more likely to visit their families as per their own terms (6.8% vs. 3.2%) or in consultation with their husbands (69.4% vs. 59.9%) than succumbing to husbands’ unilateral decision (10.0% vs. 18.7%) as compared to child-brides (χ^2^ = 31.830; α = .001). But, for both group of women, the decision to visit families is more likely to be shared than unilateral – save for some variation for husband’s share.

**Table 4:** Decision-making on family visits and procreation by age at first marriage

			Age of first marriage		Total
			< 18	≥18	
Decision Maker: Visit to wife’s family****	I, Myself	N (%)	14 (3)	57 (7)	71 (6)
	Husband	N (%)	83 (19)	83 (10)	166 (13)
	Together	N (%)	266 (60)	579 (69)	845 (66)
	Inapplicable	N (%)	81 (18)	115 (14)	196 (15)
**Total**		**N (%)**	**444 (100)**	**834 (100)**	**1278 (100)**
Decision Maker. Have a child****	I, Myself	N (%)	13 (3)	19 (2)	32 (3)
	Husband	N (%)	53 (12)	89 (11)	142 (11)
	Together	N (%)	235 (53)	538 (65)	773 (61)
	Inapplicable	N (%)	143 (32)	188 (22)	331 (25)
**Total**		**N (%)**	**444 (100)**	**834 (100)**	**1278 (100)**
Decision Maker. Use/Non-use of contraceptives****	I, Myself	N (%)	52 (12)	105 (13)	157 (12)
	Husband	N (%)	41 (9)	33 (4)	74 (6)
	Together	N (%)	257 (60)	558 (67)	815 (64)
	Inapplicable	N (%)	94 (21)	138 (16)	232 (18)
**Total**		**N (%)**	**444 (100)**	**834 (100)**	**1278 (100)**

Percent in cells is calculated *within* age at first marriage.

****sig at α = .001 (Source: Survey, 2017)

The (non) use of contraceptives is another indicator of women’s decision-making power at household level, and the results in Table 4 underline that husbands retained disproportionate power in deciding whether or not wives will use contraceptives (χ^2^ = 17.781; α = .001) or when they can have a child (χ^2^ = 21.231; α = .001) when wives’ age at first marriage was below 18. The majority of married women in both groups made shared decisions together with their husbands on both issues; but, percentage differentials between the two groups show that those married at/after 18 negotiated decisions on when to have a child (66.8% vs. 54.8%), or use contraceptives (79.5% vs. 68.6%) more often than child-brides. Note here also that those who marry at/after 18 (84%) are more likely than those who marry before 18 (79%) to have ever used contraceptives.

#### Early marriage and healthcare

With regard to receiving medical care (Table 5), statistically significant difference existed on who made decisions when wives fell ill (χ^2^ = 10.734; α = .02): most decisions were shared (55.7%) or made unilaterally by husbands (24.7%). Between the two groups, women married at/after 18 were almost twice as likely as child-brides to decide on their own to seek or receive medical services when they fell ill. On the other hand, there was no statistically significant difference between spouses on who made the decision to seek medical treatment when children were the once who fell ill. Parental decision-making powers did not differ much when it was the child’s, rather than the mother’s, wellbeing at stake.

**Table 5:** Decision-making on seeking medical care by age at first marriage

			Age of first marriage		Total
			< 18	≥18	
Decision Maker. Wife receives medical care**	I, Myself	N (%)	14 (3)	49 (6)	63 (5)
	Husband	N (%)	114 (26)	202 (24)	316 (25)
	Together	N (%)	236 (53)	476 (57)	712 (56)
	Inapplicable	N (%)	80 (18)	107 (13)	187 (15)
**Total**		**N (%)**	**444 (100)**	**834 (100)**	**1278 (100)**
Decision Maker. children receive medical care	I, Myself	N (%)	10 (2)	22 (3)	32 (3)
	Husband	N (%)	29 (7)	42 (5)	71 (6)
	Together	N (%)	197 (44)	406 (49)	603 (47)
	Inapplicable	N (%)	208 (47)	364 (44)	572 (45)
**Total**		**N (%)**	**444 (100)**	**834 (100)**	**1278 (100)**
Decision Maker. Use of ANC for mothers***	I, Myself	N (%)	40 (9)	95 (11)	135 (11)
	Husband	N (%)	24 (5)	20 (2)	44 (3)
	Together	N (%)	196 (44)	406 (49)	602 (47)
	Inapplicable	N (%)	184 (41)	313 (37)	497 (39)
Total		N (%)	444 (100)	834 (100)	1278 (100)
Decision Maker. Place of child delivery	I, Myself	N (%)	28 (6)	72 (9)	100 (8)
	Husband	N (%)	42 (10)	59 (7)	101 (8)
	Together	N (%)	185 (42)	370 (44)	555 (43)
	Inapplicable	N (%)	189 (43)	333 (40)	522 (41)
Total		N (%)	444 (100)	834 (100)	1278 (100)
Decision Maker. Children Immunized/Vaccinated*	I, Myself	N (%)	70 (16)	130 (16)	200 (16)
	Husband	N (%)	17 (4)	17 (2)	34 (3)
	Together	N (%)	150 (34)	330 (40)	480 (38)
	Inapplicable	N (%)	207 (46)	357 (43)	564 (44)
Total		N (%)	444 (100)	834 (100)	1278 (100)

Percent in cells is calculated *within* age at first marriage

***sig at α = .01; **sig at α = .05; *sig at α = .1 (Source: Survey, 2017)

There is no statistically significant difference on who decides on place of child delivery (Table 5) – i.e., whether at home or health stations – (χ^2^ = 5.070; α = .17). When it comes to mothers’ availing antenatal care (ANC), nonetheless, women married at/after 18 were more likely to decide together with their husbands (48.7% vs. 44.7%), or on their own (11.4% vs. 9.0%), than accept husbands’ unilateral decision (2.4% vs. 5.4%) as compared to child-brides (χ^2^ = 11.573; α = .009). This is, however, assuming both group of women have comparable – availability and accessibility – reproductive health facilities and services, gender-mix of health professionals (husbands prefer women health professionals to deliver their babies), etc.

Similarly, on how decisions on children’s immunization/vaccination were made at household level, there was weak statistical difference between the married women depending on their ages at first marriage. But observed differences show that child-brides were twice more likely to accept husbands’ unilateral decisions (3.8% vs. 2.0%), or less likely to share the role with their husbands (33.8% vs. 39.6%), as compared to women married at/after 18. However, cautious interpretation of this result must take into account the weak statistical association between age at first marriage and decision making on children’s immunization/vaccination (χ^2^ = 7.035; α = .071).

## Discussion

Building on the survey results, this section explores further – using narratives and discourses generated through FGDs and in-depth interviews – the main findings on the effects of early marriage on women’s empowerment in western Amhara region. The discussion is embedded within systems theory and follows similar structure of presentation as the results section.

The survey results showed that one-third of married-women in western Amhara region were affected by early marriage; and, they experience various forms of marital and family disorganizations i.e., divorce, separation, martial abuse, etc. They mostly marry older men and soon afterwards drop out of school. Education is generally ‘unthinkable’ for child-brides, FGD participants at Libo Kemkim explain:

The immediate result of early marriage is dropping out of school, if they were [still] in school at the time of marriage. Husbands want their wives to quit schooling [and become stay-at-home wives] too. If child-brides stay in school, they become persistent truants or repeat grades. More than half of them repeat grades. They do not get the necessary support they need to stay in school and be successful. They are also very much depressed and isolated from the school community and their classmates (FGD_R06).

But as child-brides get older, many grew aware of their missed opportunities due to a life imposed on them. While their age-mates be and act as they are supposed to i.e., children, they toil and serve the will of an outmoded tradition. A 16 years old child-bride in Derra *Woreda* laments,

I loved going to school and did well too …. But when I reached Grade-7, my mother started complaining why I wanted to continue going to school instead of getting married. She used to name girls in my neighbourhood who married younger than I was at the time …. Now, I’m jealous of my former classmates who still go to school and progress through grades …. I sometimes cry alone (Interview_S_1_05).

Child-brides become more and more isolated and restricted to the household as years go by. A child-bride who married at 15 and dropped out of school at 6th grade says, “I don’t see my friends frequently. They visit during weekends, since they have school during weekdays. This makes me sad and angry. Seeing them going to school with books and in uniforms, I feel sad and I want to cry” (Interview_S_2_05).

Child-brides were also more likely to experience early sexual debut and pregnancy – and probably suffer from medical complications. Childbirth effectively ends their childhood as they become child-mothers: “My brothers used to tease me about the way I carried my son around. I did not know how to do it right. But they supported me a lot in raising him” (Interview_S07).

As the survey results revealed, child-brides were more likely to sustain verbal abuse (47%) than martial rape (28%) or beating (16%) by first husbands than adult-brides. These incidents remain mostly unreported to authorities, unless they result in serious injuries – and even these may be kept as a family matter and dealt with discretely. For they are taken for granted aspects of married life or a trait of masculinity as the experience of a child-bride who married a 22-year-old man when she was 15 attests. When asked if her husband ever verbally or physical abused her, she replies, with a dismissive chuckle in her voice, “Isn’t he a man?! Of course, he swears and insults me when things are not in order at home” (Interview_S_2_05).

As patriarchal culture normalizes spouse abuse and violence, men tend to regularly use it to settle disagreements with and/or assert their authority over their wives. Mostly against child-brides, due to age gap, husbands may feel justified, or even required, to use force to ensure conformity to patriarchal norms of marital relations. In fact, survey results underlined the importance of age at first marriage whereby such scenarios are significantly reduced among women married at/after 18 – their delayed marriage gave them the time and maturity to influence the mate selection process and martial relations.

Child-brides, compared to women married at/after 18, were also consistently powerless in making or negotiating decisions with their husbands on important household matters. At best, they shared decision-making powers with their husbands, which, considering their broad definition of ‘shared’ decision-making process, may not tell us much about their real-live experiences. Furthermore, their roles in household decision-making processes varied by the activity under consideration. For instance, they were better involved when the decision is about buying groceries than children’s clothing. This is not contrary to the prevailing patriarchal norms, however, as groceries are ‘must-have’ but children’s clothing could be optional depending on other priorities, and it is on such matters that men retain the authority.

On the other hand, child-brides and adult-brides were not different regarding decisions on when and how often they visited their families. But there is more to the process than what meets the eye; and, it is related to parental approval of the union – from initiation to formation and maintenance – which puts the husband at ease when it comes to his wife visiting her parents/families. In other words, it only implies the husband temporarily transferring the locus of control from his house to her parents’. Furthermore, marriage involves the transference of rights between domestic groups, and there is always a scope for a wife to visit and contribute labour or services to her parents/family in such occasions as childbirth, pre- and post-natal care, weddings, death, etc.^4^ A husband cannot refuse his wife these socially sanctioned visits and roles without risking ridicule and contempt. But he can negotiate the length of her family visit, which exemplifies one of the few contexts where some level of negotiation (and empowering scenario) is built into marriage norms for married women.

The role of child-brides in decisions on conceiving, spacing and number of children, however, paints the usual picture of disempowerment, and it is primarily related to the cultural value that children have in the study community. As a norm, early marriage is actually marriage between families with procreation i.e., generational continuity in its core. In western Amhara, children are also seen as blessings, making the use of contraceptives immoral, sinful and threat to the foundation of traditional marriage. In the eyes of the community, children make a family complete; and oftentimes, contraceptive use is discouraged especially among young brides, which explains why this study found fewer child-brides ever using contraceptives. Hence, if and when husbands resist the use of contraceptives, they have the cultural leverage to back it up. However, those who married at/after 18 were better placed to negotiate the terms as their marriage was most likely shaped by their preference – with varying levels of parental and family involvement, of course.

Child-brides responded to these scenarios differently. Some resigned and accepted their fate, while others, like the child-bride at Bahir Dar city, revolted: “My mother married me off to a 22-years-old man when I was only 10. I moved to his parents’ house. My in-laws were very old and I had to take care of them. I did everything around the house as well …. It was killing me. One morning, I just got up and left, and came to Bahir Dar” (Interview_S04).

Parents and families almost unilaterally and ubiquitously arrange early marriage – and they draw on cultural values to justify their decisive roles. But, with early marriage being illegal, they must proceed discretely not to alert authorities – legal departments, police, the courts, education officers, teachers, etc. – and, primarily, the girl-child herself or her friends. A legal officer at Addis Zemen Woreda (South Gondar) explains:

As people become aware of the legal repercussions, many [parents] are also getting creative to evade the law and marry-off children. Now, they use social events like *Mahèber*, *Zèkèr* or birthdays as covers. This has made modern day early marriage practices largely clandestine and illusive. Detecting or reporting it is becoming difficult (FGD_R06).

With ramped-up campaigns against early marriage, girls and young women are becoming self-aware of its illegality of early marriage and their rights to education. Self-assertive girls have learned to evade this yoking institution by refusing their parents’ wishes or, when that does not work, threatening to contact authorities. This explains why many child-brides were kept in the dark about such arrangements, making their first encounter with their husbands-to-be disillusioning: “My father arranged everything. He told me who I will marry and where I will live afterwards. I never knew the person before and the first time I saw him was when we went for medical^5^ …. They said he was 20 at the time but he looked much older to me” (Interview_S_1_05).

Child-brides may accept their parents’ decisions to marry early for various reasons: to fulfil a terminally-ill parents’ wish to see their children forming a family; to escape poverty or help parents benefit from bride-wealth (*tèlosh*); to enable a family forge desirable alliance with a respected family through marriage; etc. But growing older brings opportunities of self-awareness and maturity for most child-brides. Their exposure to the world outside induces changes in their views, attitudes and behaviours – changes that test their resolve to continue respecting parents’ life-changing decisions. Husbands and parents usually treat this change as a sign of moral corruption and respond with corrective measures, abuse or violence. This explains why most child-brides are more prone to various forms of family disorganization, abuse or disempowerment compared to adult-brides.

There is a common thread in these discussions i.e., age. In Ethiopia, as in most other societies, 18 is more than just a number. It is the age of legal emancipation, which comes with the right to decide on one’s own or give independent consent to contractual agreements including marriage. However, most communities in the study area – bar for the two cities of Gondar and Bahir Dar – define girls’ readiness for marriage well below 18 – with stark contradiction to the Family Law. The Law may see the child in a girl below 18. But for people around her, she could be at ‘the right age’ to become, or start her journey to become, a good-wife and/or a good-mother.

As future household heads, on the other hand, boys are allowed to grow older, develop their life-skills, and become experienced and mature. They enjoy greater scope for experimentation and financial independence before venturing to form family. Conversely, since early childhood, girls are taught to regard marriage, family and motherhood as the good-woman’s virtues. As soon as girls’ physical development ‘catch the eye,’ the norm is for her parents to identify a suitable marriage plan. This scenario is intimately related to the gendered socialization of boys and girls in patriarchal societies like Ethiopia. A secondary school principal at Yèlma-èna-Dénsa *Woreda* concurs:

The [rural] community sees boys and girls differently. It marries girls early as protection from risks [such as rape, abduction or adolescent pregnancy as they traverse great lengths to and from school]. Moreover, parents do not have faith in girls to be successful in education and lead a decent life on their own as boys. They think marriage is the best way for girls to have a fruitful adult life. For boys, parents usual wait for them to reach their potential in education, or learn to stand on their feet. This, however, does not happen for [most] girls (FGD_R07).

Whether parents arrange marriage for their daughters depends on a unique definition of ‘an appropriate mate,’ FGD participants at Chilga *Woreda* add:

What parents and the community take into account during arranging early marriage [for a girl] is whether the groom-to-be can provide for her. They don’t consider its bad health or other effects in her life …. [As a norm,] Parents [could also agree] to give their daughter’s hands in marriage if they are convinced that a boy [or his family] is economically well and promise to let her continue her education …. But this promise rarely materializes [FGD_R01].

But to ensure a child-bride keeps a good home, she is preferred (i.e., arranged) to marry someone older with the means to provide for her and the cultural wisdom to make important decisions on household and broader matters. The arrangement works well for boys who postpone marriage till they acquire the means to provide for a family and administer its affairs.

Consequently, in a patriarchal arrangement where power lies in the hands of men and the husbands are usually older, child-brides remain structurally fixed to subservient position in their own marriages and houses. With largely ineffective systems to prevent early marriage or ensure child-brides’ safety and rights in an unlawful arrangement, husbands can easily draw on the patriarchal culture to impose their decisions, whereby consulting or involving wives becomes an indulgence they do well without. Even with changes that undermine patriarchal rules on marital relations, as Kolb and Straus argue, “individuals socialized to operate in one system of family organization may have difficulty [in] operating under new standards” [59].

## Conclusions

Informed by systems theory and using a mixed methods approach, this study compared child- and adult-brides in western Amhara region to assess their roles in household decision-making processes. It reported that child-brides are more likely to experience family and marital disorganizations – they had higher rates of both divorce and remarriage. They were also more likely to suffer from various types of abuse and violence while committed to subordinate roles in most household decision-making processes.

Systems theory teaches us that marital relations and household decision-making processes reflect the idiosyncrasies of members, the functional prerequisite of the household unit and the wider cultural milieu. As the study results revealed, women married at latter ages were able to influence household decision-making processes in ways that recognize their preferences and wellbeing. Age is not just a biological factor as it entails cultural definition of one’s scope of involvement and influence in household as well as wider sociocultural, economic and political affairs of the community. The interactions between individual and community factors seem to create better negotiation powers for women married as adults than those married as children.

Using systems theory, the discussion of results underlined the relevance of unravelling the interactions between individual, institutional and community factors to understand and/or change the power dynamics between spouses at household level. Furthermore, its findings imply that sectoral interventions will struggle to bring much-sought after emancipation of women in patriarchal institution and culture and abolish early marriage. The alignment between the study findings and the premises of system theory illustrate why child-brides faced resistance from their husbands, families and communities to be involved in household decision-making processes. There were reports about married women sustaining spousal abuse for wanting to have a say on what happens in the household. There were also women who did not want to do so since that was not how they were brought up and saw husbands’ unilateral decision-making powers as something natural.

In sum, the study results reveal that with increasing age comes physical, social and emotional maturity, and delaying marriage improved married women’s empowerment in household decision-making processes. But this change did not unfold unilaterally and in simple correlation with women’s age at first marriage since it bore the imprints of individual, institutional and cultural factors. There were instances of neglect, resistance or abuse as individuals, institutions and norms adjust to and accommodate women’s preferences and wills in marital relations and household management. We conclude by stating the obvious: if women do not have much decision-making power at the domestic sphere, which is traditionally defined as their domain, how would the gap be in the public sphere, which is traditionally out of their reach or influence? The authors believe this is one of the areas that further research could productively explore.

## Endnotes

Following the political unrest of 2018, the North Gondar Zone has been subdivided into three zones with their own administrative structures – North, Central and West zones – in 2019. But this study was conducted in 2017 – before the restructuring – and covered *Kebeles* in the then North Gondar Zone.

## Supplementary Information

**Additional file 1.**

## Data Availability

The datasets used and/or analysed during the current study are available from the corresponding author on reasonable request.

## Abbreviations

CSA: Central Statistical Authority (Ethiopia)
DHS: Demographic and Health Survey (Ethiopia)
E_4_Y: Engaged, Educated, Empowered Ethiopian Youth
FGD: Focus Group Discussion
ICRW: International Center for Research on Women
M: Mean
MoE: Ministry of Education (Ethiopia)
MoWCYA: Ministry of Women, Children and Youth Affairs (Ethiopia)
SD: Standard Deviation
SPSS: Statistical Package for Social Sciences
TGE: Transitional Government of Ethiopia
UNFPA: United Nations Fund for Population (Activities)
UNHCR: United Nations High Commissioner for Refugees
UNICEF: United Nations International Children’s Emergency Fund

## Footnotes

1 Countries with higher prevalence of early marriage are Niger (82%), Bangladesh (75%), Chad (73%), Yemen (64%), Mali (63%), Nepal (63%), Mozambique (59%) and Ethiopia (57%)

2 The Federal Democratic Republic of Ethiopia had 9 regional states (Tigray, Afar, Amhara, Oromia, Somali, Benishangul-Gumuz, Southern Nations, Nationalities and Peoples, Gambela, and Harari) and 2 city administrations (Addis Ababa and Dire Dawa) until 2020. Currently, the Sidama region had broken-off with SNNP and has been recognized as a separate region, making the number of regional states 10.

3 Following the political unrest of 2018, the North Gondar Zone has been subdivided into three zones with their own administrative structures – North, Central and West zones – in 2019. But this study was conducted in 2017 – before the restructuring – and covered Kebeles in the then North Gondar Zone.

4 Many Africans and Latin Americans practice a tradition for new moms called *la cuarentena,* a Hispanic word that to refer to a period of approximately 6 weeks, during which new mothers abstain from sex and solely dedicate their time and energy to breastfeeding and taking care of themselves and the baby. Members of the family participate to cook, clean and take care of other children, if there are any. What is different in the Ethiopian case is that pregnant women generally return to their parents’ house and stay there receiving all pre- and post-natal care by their family members. The length of the stay covers a week or days before birth and until the baby is baptized, for Christian folks.

5 Medical assessment of couples’ health status – mainly HIV/AIDS – is becoming increasingly a requirement to legalize marriage in Ethiopia.
