# Supplementary material for: Early marriage and women’s empowerment: the case of child-brides in Amhara National Regional State, Ethiopia
Source: BMC Int Health Hum Rights. 2020 Dec 14;20:30. doi: 10.1186/s12914-020-00249-5 (PMC7734808; doi:10.1186/s12914-020-00249-5)
Supplement: Supplementary file 1 — Additional file 1. [file 12914_2020_249_MOESM1_ESM.doc]

**Manuscript:** **Early Marriage and Women’s Empowerment: The case of child-brides in Amhara National regional State, Ethiopia.**

**Consolidated criteria for reporting qualitative studies (COREQ): 32-item checklist**

Developed from:

Tong A, Sainsbury P, Craig J. Consolidated criteria for reporting qualitative research (COREQ): a 32-item checklist for interviews and focus groups. *International Journal for Quality in Health Care*. 2007. Volume 19, Number 6: pp. 349 – 357

| **No. Item** | **Guide questions/description** | **Reported on Page #** |
| --- | --- | --- |
| **Domain 1: Research team and reﬂexivity** |  |  |
| *Personal Characteristics* |  |  |
| 1. Inter viewer/facilitator | Which author/s conducted the interview or focus group? | Page 26-27 |
| 2. Credentials | What were the researcher’s credentials? E.g. PhD, MD | Page 28-29 |
| 3. Occupation | What was their occupation at the time of the study? | Page 28-29 |
| 4. Gender | Was the researcher male or female? | Page 28-29 (28. Mrs. Ansha) |
| 5. Experience and training | What experience or training did the researcher have? | Page 28-29 |
| *Relationship with participants* |  |  |
| 6. Relationship established | Was a relationship established prior to study commencement? | Page 27  . |
| 7. Participant knowledge of the interviewer | What did the participants know about the researcher? e.g. personal goals, reasons for doing the research | Page 11-12 |
| 8. Interviewer characteristics | What characteristics were reported about the inter viewer/facilitator? e.g. Bias, assumptions, reasons and interests in the research topic | Page 11-12 |
| **Domain 2: study design** |  |  |
| *Theoretical framework* |  |  |
| 9. Methodological orientation and Theory | What methodological orientation was stated to underpin the study? e.g. grounded theory, discourse analysis, ethnography, phenomenology, content analysis | Page 8 |
| *Participant selection* |  |  |
| 10. Sampling | How were participants selected? e.g. purposive, convenience, consecutive, snowball | Page 8-10.  In addition, not to lengthen the methods section, Kebeles selected for the survey were not described in the manuscript. Hence, here are the names of the 4 Kebeles in each district (U=urban; R=rural):   1. Chilga Woreda (Code.01):    1. Serako (R)    2. Eyaho Seraba (R)    3. Bihona (R)    4. Aykel 02 (U) 2. Gondar Zuria Woreda (Code.02)    1. Bahri Gimb (R)    2. Lemba Arbaytu (R)    3. Sebah Gebriel (R)    4. Enfiranz (U) 3. Gondar city (Code.03)    1. Maraki Subcity (U)    2. Arbegnoch Subcity (U)    3. Mehal-Arada Subcity (U)    4. Azezo AyerMarefiya Subcity (R) 4. Bahir Dar city (Code.04)    1. Hidar 11 Subcity (U)    2. Belay Zeleke Subcity (U)    3. Gish-Abay Subcity (U)    4. Zenzelima Subcity (R) 5. Derra Woreda (Code.05)    1. Ambesame (U)    2. Ema Shenkorie (R)    3. Wochech (R)    4. Zara Michael (R) 6. Libo-Kemkim Woreda (Code.06)    1. Addis Zemen (U)    2. Ambo Meda (R)    3. Bura (R)    4. Birkutie (R) 7. Yilma-ena-Densa Woreda (Code.07)    1. Kilelet (R)    2. Dembash (R)    3. Goshye (R)    4. Adet (U) |
| 11. Method of approach | How were participants approached? e.g. face-to-face, telephone, mail, email | Page 10-11 |
| 12. Sample size | How many participants were in the study? | Page 9 |
| 13. Non-participation | How many people refused to participate or dropped out? Reasons? | Page 9 |
| *Setting* |  |  |
| 14. Setting of data collection | Where was the data collected? e.g. home, clinic, workplace | Page 9  . |
| 15. Presence of non-participants | Was anyone else present besides the participants and researchers? | Page 9-10 |
| 16. Description of sample | What are the important characteristics of the sample? e.g. demographic data, date | Page 13-14 |
| *Data collection* |  |  |
| 17. Interview guide | Were questions, prompts, guides provided by the authors? Was it pilot tested? | Page 10-11 |
| 18. Repeat interviews | Were repeat inter views carried out? If yes, how many? | No, inferred on page 10-11 |
| 19. Audio/visual recording | Did the research use audio or visual recording to collect the data? | Page 11-12 |
| 20. Field notes | Were ﬁeld notes made during and/or after the inter view or focus group? | Page 11-12 |
| 21. Duration | What was the duration of the inter views or focus group? | Page 10 |
| 22. Data saturation | Was data saturation discussed? | No. Page 9 |
| 23. Transcripts returned | Were transcripts returned to participants for comment and/or correction? | Page 12-13 |
| **Domain 3: analysis and ﬁndings** |  |  |
| *Data analysis* |  |  |
| 24. Number of data coders | How many data coders coded the data? | N.A. Page 11 |
| 25. Description of the coding tree | Did authors provide a description of the coding tree? | NA. Page 11 |
| 26. Derivation of themes | Were themes identiﬁed in advance or derived from the data? | Page 11 |
| 27. Software | What software, if applicable, was used to manage the data? | Page 11 |
| 28. Participant checking | Did participants provide feedback on the ﬁndings? | Page 12-13 |
| *Reporting* |  |  |
| 29. Quotations presented | Were participant quotations presented to illustrate the themes/ﬁndings? Was each quotation identiﬁed? e.g. participant number | Page 15-18 |
| 30. Data and ﬁndings consistent | Was there consistency between the data presented and the ﬁndings? | Yes, there was.  Page 13-23 |
| 31. Clarity of major themes | Were major themes clearly presented in the ﬁndings? | Yes. they were.  From 13-23 |
| 32. Clarity of minor themes | Is there a description of diverse cases or discussion of minor themes? | Discussion of major and minor themes  From page 13-23 |
